# Inguinal Hernia With Gastric Content

**DOI:** 10.7759/cureus.70346

**Published:** 2024-09-27

**Authors:** Danah S Alhajjaji, Mayyas A Alnajmi, Waseem M Alyamani, Rayyan Bassas, Maher A Ghazawi

**Affiliations:** 1 Medicine and Surgery, College of Medicine, Umm Al-Qura University, Makkah, SAU; 2 Radiology, Al-Noor Specialist Hospital, Makkah, SAU

**Keywords:** computed tomography, emergency general surgery, gastric, hernia, incarcerated hernia, inguinal, inguinal hernia, stomach

## Abstract

It is incredibly rare to find stomach content inside an inguinal hernia. Here, we report a 77-year-old male patient with a long-standing history of a left inguinal hernia spanning over a decade. Notably, the hernia had become irreducible for the past 20 days. CT scan of the abdomen and pelvis revealed a substantial left inguinal hernia extending into the left scrotal region causing bowel obstruction. The hernial neck measured approximately 5.5 cm in transverse diameter. Protrusion through this defect included the stomach, small and large bowel loops, and free mesenteric fat and vessels into the hernial sac. The patient underwent a life-saving exploratory laparotomy and the hernial sac was reduced and repaired. In conclusion, inguinal hernias are common, but stomach content cases are extremely rare and they usually present with gastric outlet obstruction or gastric perforation. CT is recommended to visualize the stomach within the hernia and to exclude complications. Surgical repair is usually the management of choice.

## Introduction

Indirect inguinal hernia, the most common form of hernia, presents as a bulge in the groin near the upper part of the inner thigh. It is caused by fat or a portion of the intestines entering the inguinal canal by pushing through a weak point in the abdominal wall that surrounds it [1]. Hernia is most prevalent in adult men over 50 years old at more than 75%, with approximately one-third of men being diagnosed with an inguinal hernia at some point in their lives [2]. The omentum or nearby movable structures, such as the small or large intestines, are typically present in inguinal hernias, with the presence of stomach contents being a particularly rare presentation. This type of presentation could be related to chronic increased intra-abdominal pressure or a congenital weakness that causes progressive incarceration of the colon and intestines, which pulls the stomach inward and into the hernia sac [3].

Previous studies have reported a limited number of cases of inguinal hernia containing the stomach [3-10], with none reporting the condition in a patient in Saudi Arabia. In this report, we present a case of a 77-year-old male with left inguinal hernia containing the stomach.

## Case presentation

The case presented here involved a 77-year-old male patient with a long-standing history of left inguinal hernia spanning over a decade. Despite the chronic nature of the hernia, the patient did not seek medical attention until presenting with complaints of abdominal pain, nausea, and coffee ground vomiting. Notably, the patient’s hernia had been irreducible for the past 20 days. The patient also reported unintentional weight loss over the past month, although specific measurements were not recorded. The absence of changes in bowel habits, urinary symptoms, or other constitutional symptoms is an intriguing aspect of this case.

The patient denied having any chronic illnesses or prior surgeries, emphasizing the continuing nature of the left inguinal hernia, which extended to the scrotum. The symptoms of abdominal pain, nausea, and coffee ground vomiting suggested a potential complication related to the hernia. The prolonged irreducibility added complexity to the clinical picture. Despite unintentional weight loss, the patient had not documented the extent or specifics of the weight changes.

A physical exam determined the patient to be conscious and oriented but in pain and dehydrated, although not in acute distress. Tenderness was noted in the left inguinal region. A substantial left inguinal hernia was observed with bowel palpable in the scrotum. Laboratory test result parameters were within normal ranges except for mild anemia and an elevated white blood cell count of 16, as shown in Table 1, indicating a potential inflammatory or infectious process.

**Table 1:** Laboratory test results.

Test	Patient result	Reference range
Hemoglobin	10	11-14 g/dL
White blood cells	16 x 10^9/L	4.5-11 x 10^9/L
Platelets	217 x 10^9/L	150-450 x 10^9/L

**Figure 1:**
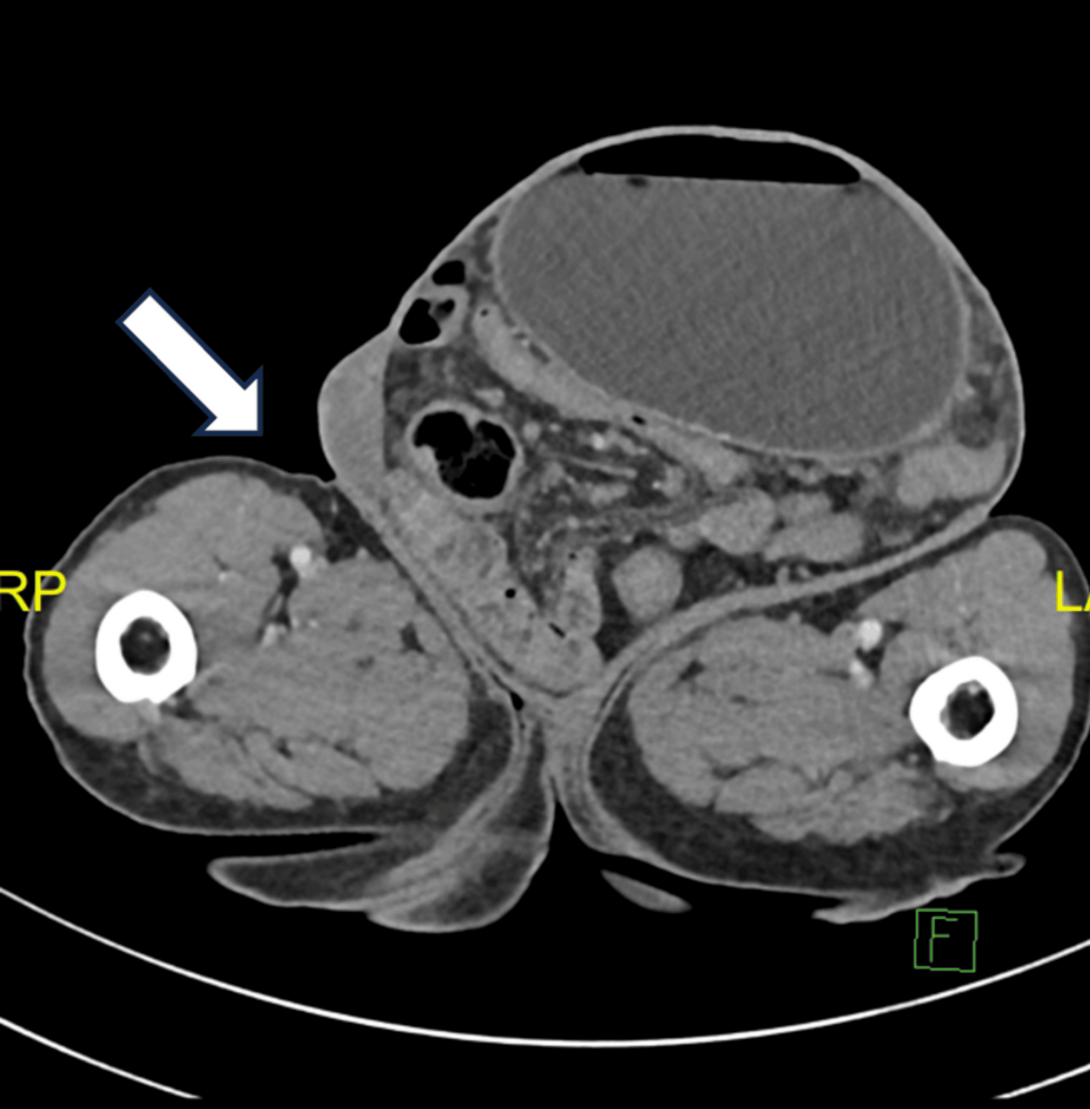
Computed tomography of the abdomen and pelvis showing dilated stomach within the left inguinal hernia sac.

**Figure 2:**
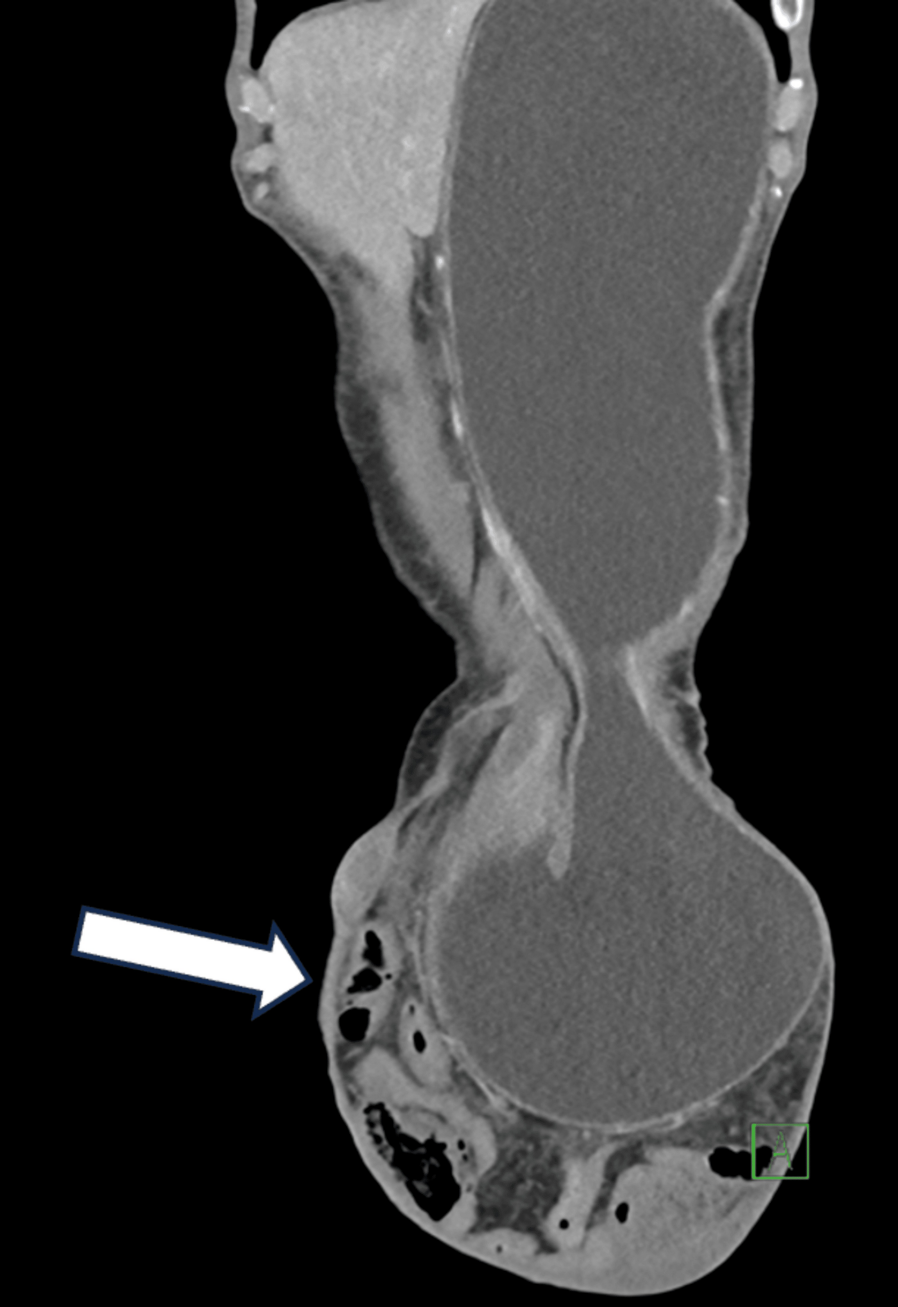
Computed tomography of the abdomen and pelvis coronal cross-section showing dilated stomach extending into the left inguinal hernia.

A CT scan of the abdomen and pelvis revealed a substantial left inguinal hernia extending into the left scrotal region. The hernial neck measured approximately 5.5 cm in transverse diameter, while the hernial sac measured 17 × 18 × 21 cm (anteroposterior, transverse, and craniocaudal, respectively). Protrusion through this defect included the stomach, loops of the small and large bowel, and free mesenteric fat and vessels into the hernial sac causing small bowel obstruction. Significantly, the stomach was distended to the pylorus, indicating possible gastric outlet obstruction. A transitional zone was identified at the pylorus level. Despite these findings, wall enhancement and the thickness of afferent and efferent bowel loops and the stomach were preserved. Mild free fluid was present, but there was no evidence of free air, pneumoperitoneum, pneumatosis intestinalis, or submucosal hemorrhage. The herniated small bowel appeared to collapse (Figures 1, 2).

Due to the findings of the small bowel and gastric outlet obstruction, the patient was shifted to the OR for a life-saving exploratory laparotomy with a narrowing of the wide left inguinal internal ring. OR findings included a herniated largely dilated stomach with 3200 ml of gastric content suctioned out and band at the pylorus of the stomach that was released and a herniated collapsed viable large and small bowel and band at gubernaculum with sigmoid that was released. The surgical team planned for staged surgery with anatomical reinforcement and mesh placement on an elective basis due to the long-standing herniation.

## Discussion

Inguinal hernias are the most common type of abdominal wall hernias [11]. They typically contain various combinations of bowel, omentum, portions of the urinary bladder, ovaries, and uterus [12]. It is rare for the contents of an inguinoscrotal hernia to include the pancreas due to it being a retroperitoneal organ or the stomach due to its fixation in the upper abdomen by the gastrohepatic, gastrosplenic, gastrocolic, and gastrophrenic ligaments. Although the exact cause of the descent of the stomach into the scrotum is unknown, it is thought that this movement is caused by long-standing traction on its attachments. Patients with inguinoscrotal hernia with stomach content predominantly present with gastric perforation, gastric outlet obstruction, or bowel ischemia [8].

A systematic review (2021) of 21 cases of inguinoscrotal hernias with gastric contents found that all cases were male patients, with a median age of 71 years old, and the laterality of the stomach commonly being on the left side of the groin (67%, n = 14). The most common presenting symptoms were abdominal pain and vomiting (67%, n = 14). Management approaches varied based on whether the case presentation included gastric perforation or gastric outlet obstruction. Surgical approaches were taken for all patients presenting with gastric perforation, but surgical techniques varied. Most patients (eight out of 10) were treated with combined surgery with midline laparotomy and an incision in the groin. Two patients were offered single operative management, one of which received a midline laparotomy and the other a laparoscopic repair. Management of the care of patients presenting with gastric outlet obstruction varied, with conservative management used to treat five patients successfully and the remaining four treated operatively [6].

## Conclusions

In conclusion, while inguinal hernias are common, stomach content cases are extremely rare, and they usually present with gastric outlet obstruction or gastric perforation. Computed tomography is recommended to visualize the stomach within the hernia and to exclude complications. In the case presented here, surgical management was preferable due to complications, while conservative management is considered appropriate in patients with comorbidities.
